# Characterization of BAS-1, a novel class A β-lactamase encoded in *Bacteroides thetaiotaomicron*

**DOI:** 10.1128/aac.00391-26

**Published:** 2026-06-15

**Authors:** María Aznar Fernández, María José Gómez Gómez, José Miguel Cisneros, José Antonio Lepe, José Manuel Ortiz de la Rosa

**Affiliations:** 1 Clinical Unit of Infectious Diseases, Microbiology and Parasitology, University Hospital Virgen del Rocío 16885, Seville, Spain; 2 Institute of Biomedicine of Seville (IBiS), University Hospital Virgen del Rocío/CSIC/University of Seville, Seville, Spain; 3 Centro de Investigación Biomédica en Red de Enfermedades Infecciosas (CIBERINFEC) 637284 , Madrid, Spain; 4 Department of Medicine, Faculty of Medicine, University of Seville 73063, Seville, Spain; 5 Department of Microbiology, Faculty of Medicine, University of Seville 73063, Seville, Spain; University of Fribourg, Fribourg, Switzerland

**Keywords:** *Bacteroides thetaiotaomicron*, *bla*_BAS-1_, novel β-lactamase, resistance mechanisms

## Abstract

We characterized a novel class A β-lactamase (BAS-1) in a pan-β-lactam-resistant *Bacteroides thetaiotaomicron* clinical isolate. Whole-genome sequencing identified *bla*_BAS-1_ within a mobile genetic element associated with IS*4351*. Cloning, susceptibility testing, β-lactam inactivation method, and half-maximal inhibitory concentration assay confirmed resistance to penicillins, cephalosporins, monobactams, and reduced susceptibility to the β-lactamase inhibitors. To date, no class A β-lactamase from *Bacteroides* has been shown to display such a resistant profile, challenging the assumed reliability of β-lactam/β-lactamase inhibitor combinations.

## INTRODUCTION

Although some bacteria naturally produce β-lactamases, the main concern is their mobilization, especially ESBLs and carbapenemases (1, 2). *Bacteroides* spp., key gut microbiota members and occasional opportunistic pathogens, are recognized reservoirs of antimicrobial resistance genes (3, 4). First-line therapies (metronidazole, piperacillin–tazobactam, and meropenem) are seeing rising resistance from overuse (5–7), with more isolates carrying resistance genes, along with novel resistance determinants reported recently in such species (8–12). Here, we characterize a novel inhibitor-resistant class A β-lactamase from a highly resistant *Bacteroides thetaiotaomicron* (BT736).

**Fig 1:**
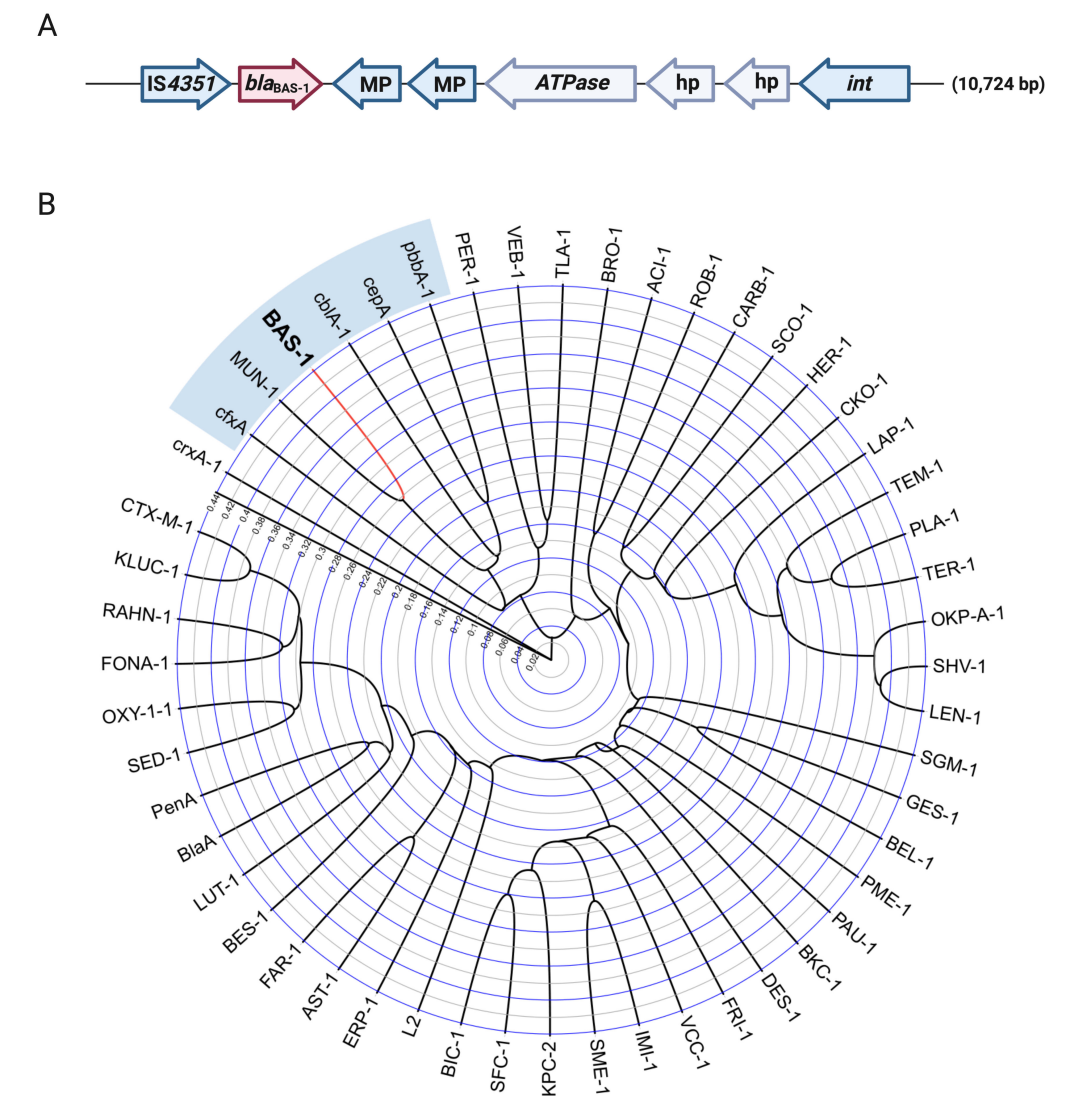
(**A**) Schematic representation of the genetic environment surrounding the *bla*_BAS-1_ resistance gene on the *B. thetaiotaomicron* BT736 isolate. Genes and their corresponding transcription orientations are indicated by colored arrows. The red, blue, and gray arrows indicate antimicrobial resistance genes, insertion sequences/transposases/mobilization genes, and other genes, respectively. (**B**) Phylogenetic tree of amino acid sequences of representative β-lactamases found in the bacterial realm including the BAS-1 β-lactamase (in bold).

Among a collection of *Bacteroides* spp. isolates recovered over a 6-month period at Virgen del Rocío University Hospital, a pan-β-lactam-resistant *B. thetaiotaomicron* (BT736) was identified from an abdominal abscess collected in March 2023 from a 74-year-old male admitted for surgical intervention who received amoxicillin/clavulanic acid during his hospitalization (Table 1). In order to know the resistance mechanisms, whole-genome sequencing was performed through Oxford Nanopore Technologies, and the data obtained were curated and analyzed as previously described (Tables S2 and S3) (13). Although the species of the clinical isolate was identified by MALDI-TOF, taxonomic confirmation was performed by comparison with the reference *Bacteroides thetaiotaomicron* DSM 2079, yielding an average nucleotide identity of 97.81%. Regarding its resistome, the BT736 genome (GenBank accession number: PRJNA1436858) carried *tet(X*), *tet(Q*), *ermF*, and *cfxA*. Considering that *cfxA* was the only β-lactamase detected, and given that it has been described as conferring resistance to penicillins and cephalosporins while remaining susceptible to β-lactamase inhibitors (14), the observed resistance to carbapenems and β-lactam/β-lactamase inhibitor combinations cannot be explained by CfxA alone. Therefore, a β-lactam inactivation method (β-LIM) was performed on the BT736 clinical isolate, as previously described (15). The β-LIM result showed no hydrolysis of carbapenems, indicating that carbapenem resistance was likely mediated by reduced permeability and/or efflux pump activity (data not shown). On the other hand, resistance to β-lactam/β-lactamase inhibitor combinations suggested the presence of an inhibitor-resistant β-lactamase hydrolyzing all β-lactams except carbapenems. Then, resistome reanalysis was performed through lower coverage and identity thresholds, revealing the presence of an open reading frame encoding a novel class A β-lactamase (*bla*_BAS-1_), whose predicted protein showed 64% identity and 100% coverage with *bla*_MUN_ at the amino acid level, according to BLASTp analysis against the NCBI and β-lactamase databases (10, 16, 17). BLASTp also identified a partial protein sequence in *Bacteroides* spp. (MBP6170604.1) with 71% coverage and 100% identity, as well as another protein in a genome classified as *Candidatus Cryptobacteroides avistercoris* (MBO8480007.1) with 98% coverage and 91.13% identity. This identity level supports classifying BAS-1 as a distinct β-lactamase, not a variant of known enzymes. To identify other proteins closely related to BAS-1, a phylogenetic tree was constructed using the most representative class A β-lactamases with the Interactive Tree of Life tool (18). This analysis showed that the novel β-lactamase clustered with previously described β-lactamases (CfxA, MUN-1, CblA-1, CepA, and PbbA) from *Bacteroides* spp. (Fig. 1B) (10, 19–22). However, when analyzed at the functional group level, BAS-1 exhibited similarity to CfxA and CblA, displaying the three conserved motifs SVFK, SDN, and KTGS (Fig. S1) (23).

**TABLE 1:** Minimal inhibitory concentrations (MICs) of the BT736 clinical isolate, *Escherichia coli* TOP10 with BAS-1, *E. coli* TOP10, and *E. coli* TOP10 with empty-pTOPO plasmid

MIC (mg/L)				
	BT736	*E. coli* TOP10 + BAS- 1	*E. coli* TOP10	*E. coli* TOP10 + pTOPO
Ampicillin	**>256**	**>256**	4	4
Amoxicillin	**>256**	**>256**	4	4
Amoxicillin–clavulanic acid	**>256**	**48**	4	4
Piperacillin	**>256**	**>256**	2	2
Piperacillin–tazobactam	**>256**	**16**	1	2
Cefazolin^*a*^	**6 mm**	**10 mm**	25 mm	26 mm
Cefuroxime	**>256**	4	4	4
Ceftazidime	**>256**	**1.5**	0.25	0.25
Ceftazidime–avibactam	**>256**	**1**	0.25	0.25
Cefotaxime	**>32**	0.03	0.03	0.03
Ceftolozane–tazobactam	**>256**	0.5	0.25	0.25
Cefepime	**>256**	**1**	0.03	0.03
Ceftaroline	**>256**	**4**	0.06	0.06
Aztreonam	**>256**	**0.25**	0.06	0.06
Imipenem	**8**	0.25	0.25	0.25
Meropenem	**8**	0.03	0.03	0.015
Ertapenem	**8**	0.03	0.015	0.015

^*a*^ Cefazolin susceptibility was tested by disk diffusion, and the results represent the diameter of the inhibition zone in mm. Bold values showed resistance or increased MIC values in comparison with the reference strain.

Based on these results, BAS-1 appears to represent a new subgroup of Ambler class A β-lactamases identified in *Bacteroides* (23). Moreover, genomic analysis identified an IS*4351* element located upstream of *bla*_BAS-1_ within a 10,724 bp mobile genetic element present at two distinct genomic locations (Fig. 1A). The IS*4351* has been linked to transposition of resistance determinants such as *erm(F*) and *tet(X*) in *Bacteroides* spp. (24) and characterized as an insertion sequence involved in the upregulation of antibiotic resistance genes. Therefore, its upstream placement could enhance *bla*_BAS-1_ expression (25).

To determine the exclusive contribution of BAS-1 production to the resistance phenotype of the bacteria, the *bla*_BAS-1_ gene was amplified using the BAS-1 F (5′-GCAAGTTGAATTCAGCAAAC-3′) and BAS-1 R (5′-TGGCTCAAAATATGTCGGGT-3′) primers and subsequently cloned into the pTOPO plasmid by using Zero Blunt TOPO PCR Cloning Kit following the manufacturer’s instructions (Thermo Fisher Scientific, USA). MICs were measured for BAS-1-producing *Escherichia coli* TOP10, *E. coli* TOP10, and *E. coli* TOP10 with empty-pTOPO plasmid. The expression of *bla*_BAS-1_ in *E. coli* TOP10 produced an increase in MICs against penicillins, cephalosporins, and monobactams, compared to the control strain. Importantly, MICs for the β-lactam/β-lactamase inhibitor combinations amoxicillin–clavulanate, piperacillin–tazobactam, and ceftazidime–avibactam remained unchanged or were not able to recover the susceptibility upon *bla*_BAS-1_ expression, indicating that the enzyme confers resistance to both β-lactams and probably to the β-lactamase inhibitors (Table 1). As shown by the results above, cloning *bla*_BAS-1_ into *E. coli* TOP10 conferred resistance to multiple β-lactams but only partially reproduced the BT736 clinical phenotype (Table 1). Previous studies attribute such discrepancies to genus-specific expression and physiological differences (26, 27). Key incompatibilities include distinct promoter motifs (*Bacteroides* −7/−33 vs *E. coli* −10/−35), divergent ribosome-binding sites that limit translation of foreign genes, and the absence in *E. coli* of required post-translational modifications or cellular conditions for full activity (26, 28, 29).

To decipher contributions of CfxA and BAS-1 to the inhibitor-resistance phenotype, we performed a modified β-LIM in the presence of the different inhibitors (Tables S1 and S2) at EUCAST-recommended concentrations, using 60 μg cefazolin disks diffusion in 1 mL culture medium with an overnight incubation under anaerobic conditions (Table S2) (30). Because BT736 carried *cfxA2*, we included the BT162 (harboring *cfxA2* as its sole resistance determinant) as a suitable comparator. To confirm that BT162 is an appropriate control, we therefore tested whether BT736 shows increased expression of *cfxA2* relative to BT162. Although CfxA has been described as being sensitive to β-lactamase inhibitors, increased expression of a β-lactamase may contribute to reduced susceptibility to β-lactamase inhibitors, even when the enzyme itself is considered inhibitor-sensitive (15, 31). RNA isolation and RT-qPCR quantified *cfxA2* and *bla*_BAS-1_ using specific primers (16S reporter gene [F: TGCATGGYTGTCGWCAGCT, R: TGMKGTYTTGACGTCRTCC], *bla*_BAS-1_ gene [F: CTTTTGTTGCACACTGGCAT, R: GGTTACAGCCACATGAAATT], and *cfxA* gene [F: CGTAGTTTTGTGTATAGCTTTAG, R: TTTGAGAAATGCTATCAGTCAA]) under previously described conditions (15). The Ct values were analyzed by the 2^−ΔΔCT^ method (32). Relative expression showed higher *cfxA2* expression in BT162, indicating that the result observed in the β-LIM with inhibitors in BT736 is unlikely due to *cfxA2* overexpression (Fig. S2). The β-LIM was positive for BT736 in the presence of all inhibitors, whereas BT162 produced inhibition zones comparable to the sterile control when inhibitors were present, suggesting CfxA2 is susceptible to β-lactamase inhibitors while BAS-1 could be associated with broad inhibitor resistance (Table S1). However, the previous assay reflects not only the activity of BAS-1 but also the influence of the bacterial background, including possible permeability defects that may limit inhibitor entry. To better define the intrinsic inhibitor resistance of BAS-1, we therefore determined the half-maximal inhibitory concentration (IC_50_) values of clavulanate, tazobactam, and avibactam using the crude extract of the BAS-1 clone and CENTA as substrate, following a previously described protocol (33). The IC_50_ values for these inhibitors were close to 1 μM (Table S4), indicating that BAS-1 is more resistant to β-lactamase inhibitors than several clinically relevant enzymes, including TEM, CTX-M, SHV, VEB, and BEL, but remains more susceptible than GES-5, GES-6, KPC, and PER enzymes (33, 34). These results suggest that the resistance phenotype observed in clinical isolate BT736, particularly to β-lactam/β-lactamase inhibitor combinations, may result from the combined effect of BAS-1 and reduced permeability of the isolate.

The characterization of a novel mobilizable class A β-lactamase in *Bacteroides* spp. has significant clinical and epidemiological implications. Encoded within a mobile element associated with IS*4351*, it confers resistance to β-lactams and reduced susceptibility to the β-lactamase inhibitors, potentially undermining the reliability of β-lactam/β-lactamase inhibitor combinations currently used as first-line therapy for anaerobic infections. These findings highlight the need for ongoing surveillance, molecular characterization, and antimicrobial stewardship.

## SUPPLEMENTAL MATERIAL

Supplemental material aac.00391-26-s0001.pdf Tables S1 to S4; Fig. S1 and S2.

ASM does not own the copyrights to Supplemental Material that may be linked to, or accessed through, an article. The authors have granted ASM a non-exclusive, world-wide license to publish the Supplemental Material files. Please contact the corresponding author directly for reuse.
