# Supplementary material for: Characterization of BAS-1, a novel class A β-lactamase encoded in Bacteroides thetaiotaomicron
Source: Antimicrob Agents Chemother. 2026 Jun 15;70(8):e00391-26. doi: 10.1128/aac.00391-26 (PMC13436384; doi:10.1128/aac.00391-26)
Supplement: Supplementary file 1 [file aac.00391-26-s0001.pdf]

Supplementary Table 1. Results of β-lactam inactivation method in combination with inhibitors

	W/O INB	CLA	TZB	AVI	VAB	REL	ENTZ
W/O bacteria	16.5	16	17	17	17	18	16
BT162	**6**	16	15.5	15	16	17	15
BT736	**6**	**6**	**6**	**6**	**6**	**6**	**6**

CLA: clavulanic acid; TZB: tazobactam; AVI: avibactam; VAB: vaborbactam; REL: relebactam; ENTZ: enmetazobactam; INB: inhibitor; W/O: without; Diameter zone expressed in mm

A)

Amino acid sequences

```
BAS-1       -----------M-RNIILPMILCFCC-TLA--------------YPQNDT--LEIRKIIQ    31
MUN-1       MKQKVPLSKMNM-TKTLLSVMLCCCCSIMA--------------FGQNMT--QEIEKIIK    43
CblA-1      ---------MKA-YFIAILTLFTCIATVVR--------------AQQMSELENRIDSLLN    36
CepA-1      ---------MQK-RLIHLSIIFFLLCPALV--------------VAQNSPLETQLKKAIE    36
PbbA-1      ----------------------------------------------MNSLRDEILQSII    13
CfxA-1      ------MEKNRKKQIVVLSIALVCIF-ILVFSLFHKSATKDSANPPLTNVLTDSISQIVS    53
                                                          : . :

BAS-1       DKQAEVGVAVLYK-D-KVYTLSNNKKYPLMSVFKFHVAVTALKKMEKENIALDSMVYIEP    89
MUN-1       GKQASVGVAIIHN-D-DIIAIANEDKYPTMSVFKFHIAVTALKKMEAENIPLDKMVYIKQ    101
CblA-1      GKKATVGIAVWTDKG-DMLRYNDHVHFPLLSVFKFHVALAVLDKMDKQSISLDSIVSIKA    95
CepA-1      GKKAEIGIAVIIDGQ-DTITVNNDIHYPMMSVFKFHQALALADYMHHQKQPLETRLLIKK    95
PbbA-1      KYDAKIGVAVMINDT-DTLTVNNCDKYPMMSVFKFHQALAVCDYMQKNGIALDSVLNISK    72
CfxA-1      ACPGEIGVAVIVNNR-DTVKVNNKSVYPMMSVFKVHQALALCNDFDNKGISLDTLVNINR    112
               . :*:*:      .         :*  ****.* *:   . ..     *:  : :.

BAS-1       RQMHANTYSPLRDKYPGQRIHISYSDIIRYTVSHSDNNTCDWLIGFVGGIEEVDAYIKSI    149
MUN-1       KEMLKNTYSPLRDKYPDQGIRISYRDIIKYTVSISDNNTCDWLIRFVGGIDKVDSYIKSL    161
CblA-1      SQMPPNTYSPLRKKFPDQDFTITLRELMQYSISQSDNNACDILIEYAGGIKHINDYIHRL    155
CepA-1      SDLKPDTYSPLRETYPQGGIEMSIADLLKYTLQQSDNNACDILFNYQGGPDAVNKYLHSL    155
PbbA-1      DELRENTYSPLRDRYPEGNFGISISELMKYTLQMSDNNACDILFNRILSVKDTDKYIRSL    132
CfxA-1      DKLDPKTWSPMLKDYSGPVISLTVRDLLRYTLTQSDNNASNLMFKDMVNVAQTDSFIATL    172
             .:  .*::*: . :    : :   ::::*::  *** :.: ::    .    . ::  :

BAS-1       G-INAQNLTETEASMHENIMCCYNNWTTPLAVAELLKKVYTEAVL-PDEYFRFLEKAMLD    207
MUN-1       G-IKDMNFTETEESMHTDIMLCYNNWSTPLAIAQLLKKLHTENIL-TKEHFAFLETAMLD    219
CblA-1      S-IDSFNLSETEDGMHSSFEAVYRNWSTPSAMVRLLRTADEKELFSNKELKDFLWQTMID    214
CepA-1      G-IRECAVIHTENDMHKNLEFCYQNWTTPLAAAKLLEIFRNENLF-DKEYKNFIYQTMVE    213
PbbA-1      G-IDDFSIEMDENDMHENLDNCYSNWTTPMAAVNLMNLFLDGDAV-SGEYYDFIYRTMTE    190
CfxA-1      IPRSSFQIAYTEEEMSADHNKAYSNYTSPLGAAMLMNRLFTEGLI-DDEKQSFIKNTLKE    231
                   .   *  *  .    * *:::  . . ::.       .       ::   : :

BAS-1       CSTGTDKLKAG-LPT-GVRFGHKTGSSDRTEMGIKIGDGDAGVIYMPDGSKCYLVVIIKD    265
MUN-1       CVSGKNKLIAG-LPT-DIKFGHKTGRSNRTADGIQISEGDAGVIYLPNGEKCYIVVLIKD    277
CblA-1      TETGANKLKGM-LPA-KTVVGHKTGSSDRNADGMKTADNDAGLVILPDGRKYYIAAFVMD    272
CepA-1      CQTGQDRLIAPLLDK-KVTMGHKTGTGDRNAKGQQIGCNDIGFILLPDGHAYSIAVFVKD    272
PbbA-1      CTTGTARLPYPLKNT-GAIIGHKTGTGDKNSDGRYIGINDIGFIDLPNGDRYLIAVLVKD    249
CfxA-1      CKTGVDRIAAPLLDKEGVVIAHKTGSGYVNENGVLAAHNDVAYICLPNNISYTLAVFVKD    291
              :*  ::           ..**** .  .  *   . .* . : :*:.    :..:: *

BAS-1       SKETDAENAKIMENIASAVYDSLK---------   289
MUN-1       SRESDDDNAKIMADISNIVYRHLNK--------   302
CblA-1      SYETDEDNANIIARISRMVYDAMR---------   296
CepA-1      SEADNRENSEIIAEISRIVYEYVTQQID-----   300
PbbA-1      SAEDYETTERIIAEISSMVYKNISNQ-------   275
CfxA-1      FKGNKSQASQYVAHISAVVYSLLMQTSVKS---   321
                       :  ::  .*
```

B)

Nucleotide sequences

```
blaBAS-1        ---------------------------------------------ATGAGAAACATTATT      15
blaMUN-1        ATGAAAC---------AAAAAGTT---CCACTATCAAAAATGAACATGACAAAAACGCTA      48
cblA-1          ---------------------------------------------ATGAAAGCATATTTC      15
cepA-1          ---------ATGCAAAAAAGACTT------------------ATACATTTATCCATTATC      33
pbbA-1          ---------ATGA------AACTA------------------AAATCAGTTGCTATTTTA      27
cfxA-1          ATGGAAAAAAACAGAAAAAAACAAATCGTAGTTTTGAGTATAGCTTTAGTTTGCATTTTC      60
                                                                          *
blaBAS-1        C---TTCCAATGATATTGTGCTTTTGTT------GCACACTGGCATACCCTCAAAAT---      63
blaMUN-1        TTATCAGTTATGCTTTGTTGCTGTTGCA------GCATTATGGCTTTCGGGCAGAAC---      99
cblA-1          ATCGCCATACTTACCTTATTCACTTGTA------TAGCTACCGTCGTCCGGGCGCAGCAA      69
cepA-1          TTCTTTCTGCTATGTCCTG---------------CCCTGGTAGTTGCGCAGAACAGTCC-      77
pbbA-1          TT------GTTGGTTTTTG---------------CCTCTGTTGTCTCAAATGCCAATTCC      66
cfxA-1          ATCTTGGTATTTTCATTGTTCCATAAATCAGCGACAAAAGATAGCGCAAATCCTCCTTTA      120
                          *
blaBAS-1        ---------GATACATTAGAAATAAGAAAAATCATTCAAGACAAACAAGCTGAGGTTGGT      114
blaMUN-1        ---------ATGACCCAAGAAATTGAAAAGATTATCAAAGGGAAGCAAGCGTCTGTCGGG      150
cblA-1          ATGTCTGAACTTGAAAACCGGATTGACAGTCTGCTCAATGGCAAGAAAGCCACCGTTGGT      129
cepA-1          --------TCTTGAAACTCAACTCAAGAAAGCCATAGAAGGGAAAAAAGCCGAAATAGGA      129
pbbA-1          TTGAACTCATTAAGAGATGAAATCCTACAAAGTATCATAAAATACGATGCTAAGATTGGG      126
cfxA-1          ACAAATGTTTTGACTGATAGCATTTCTCAAATTGTCTCAGCTTGTCCTGGCGAAATTGGT      180
                                      *           *             *      * **
blaBAS-1        GTAGCTGTTT---TATACAAAGACAAAGTTTACACTTTATCCAATAACAAAAAATACCCT      171
blaMUN-1        GTAGCAATTA---TCCATAATGACGATATAATTGCCATTGCCAACGAGGACAAATATCCC      207
cblA-1          ATAGCCGTATGGACAGACAAAGGAGACATGCTCCGGTATAACGACCATGTACACTTCCCC      189
cepA-1          ATTGCAGTCATTATCGACGGGCAAGATACGATAACAGTCAATAATGATATTCATTATCCC      189
pbbA-1          GTAGCAGTTATGATAAACGATACTGATACACTGACTGTGAATAATTGCGATAAATATCCT      186
cfxA-1          GTGGCGGTTATTGTTAATAACAGAGATACGGTTAAGGTCAATAATAAGAGTGTTTATCCT      240
                 * **  *        *        *                 *          *  **
blaBAS-1        TTGATGAGTGTTTTCAAATTTCATGTGGCTGTAACCGCATTGAAAAAAATGGAAAAGGAA      231
blaMUN-1        ACTATGAGTGTATTCAAATTCCATATAGCAGTGACAGCTCTGAAAAAAATGGAAGCAGAG      267
cblA-1          TTGCTCAGTGTATTCAAATTCCATGTGGCACTGGCCGTACTGGACAAGATGGATAAGCAA      249
cepA-1          ATGATGAGTGTTTTCAAATTTCATCAGGCATTGGCATTGGCCGATTACATGCATCATCAA      249
pbbA-1          ATGATGAGTGTCTTCAAGTTTCATCAGGCATTAGCTGTATGTGACTACATGCAGAAGAAC      246
cfxA-1          ATGATGAGTGTGTTTAAGGTTCATCAGGCATTAGCTCTTTGTAATGACTTTGACAATAAA      300
                    * ***** ** **  * ***   **  *  *        *  *  *  *     *
blaBAS-1        AATATTGCATTGGATAGTATGGTATATATTGAACCCAGACAAATGCATGCAAATACATAC      291
blaMUN-1        AATATTCCGCTGGACAAAATGGTGTATATCAAACAGAAAGAAATGCTGAAAAACACGTAT      327
cblA-1          AGCATCAGTCTGGACAGCATTGTTTCCATAAAGGCATCCCAAATGCCGCCCAATACCTAC      309
cepA-1          AAGCAACCTTTGGAAACCCGATTATTGATTAAAAAGTCGGATTTAAAGCCGGACACCTAT      309
pbbA-1          GGAATAGCCTTGGATTCAGTCCTGAATATCTCAAAAGATGAGCTTAGGGAGAATACTTAC      306
cfxA-1          GGAATTTCACTTGATACCTTAGTAAATATAAATAGGGATAAACTTGACCCAAAGACTTGG      360
                          * **        *    **           *  *        * ** *
blaBAS-1        AGTCCGTTAAGGGACAAATACCCCGGACAAAGAATACATATATCGTATAGCGATATAATA      351
blaMUN-1        AGCCCGTTAAGGGATAAATATCCCGACCAAGGGATTCGTATATCTTATAGGGATATAATC      387
cblA-1          AGCCCCCTGCGGAAGAAGTTTCCCGACCAGGATTTCACGATTACGCTTAGGGAACTGATG      369
cepA-1          AGTCCGCTTCGAGAAACATACCCGCAGGGAGGAATCGAAATGAGCATTGCCGATCTACTG      369
pbbA-1          AGTCCTTTGCGCGACCGATATCCGGAAGGAAACTTCGGAATAAGCATATCTGAACTGATG      366
cfxA-1          AGTCCTATGCTGAAAGATTATTCAGGGCCAGTCATATCATTGACAGTGAGAGATTTGCTG      420
                ** **  *     *    *   *           *     *          **  *  *
blaBAS-1        AGGTACACCGTAAGTCATAGCGACAACAATACATGTGATTGGCTCATAGGCTTTGTAGGA      411
blaMUN-1        AAATACACAGTATCGATTAGCGACAACAACACATGCGATTGGTTAATAAGGTTTGTTGGT      447
cblA-1          CAATACAGCATTTCCCAAAGCGACAACAATGCCTGCGACATCTTGATAGAATATGCAGGA      429
cepA-1          AAATATACGCTTCAGCAAAGTGACAATAATGCCTGCGATATTCTTTTTAATTATCAAGGT      429
pbbA-1          AAATACACTCTCCAGATGAGTGACAATAATGCCTGCGACATTCTGTTCAACCGTATTCTC      426
cfxA-1          CGTTATACTCTTACTCAGAGTGACAACAATGCAAGCAACCTTATGTTTAAGGATATGGTT      480
                   ** *   *       ** ***** **  *  *  *     *  *      *
blaBAS-1        GGGATAGAAGAAGTGGATGCATATATTAAATCCATTGGAATTAATGCGCAG---AACCTA      468
blaMUN-1        GGTATTGATAAAGTGGACTCCTACATAAAATCTCTTGGGATAAAGGATATG---AATTTC      504
cblA-1          GGCATCAAACATATCAATGACTATATCCACCGGTTGAGTATCGACTCCTTC---AACCTC      486
cepA-1          GGTCCGGATGCCGTGAATAAGTATCTTCATTCATTGGGAATTCGTGAATGT---GCTGTC      486
pbbA-1          AGTGTAAAAGATACTGACAAATATATCCGTTCATTGGGTATTGATGATTTT---TCAATA      483
cfxA-1          AATGTCGCTCAAACAGATAGTTTTATAGCCACACTCATTCCTCGTTCAAGTTTTCAGATA      540
                                *    *   *        *                       *
```

```
blaBAS-1        ACAGAAACAGAAGCCAGTATGCATGAAAATATAATGTGCTGCTATAATAATTGGACTACC     528
blaMUN-1        ACGGAGACAGAGGAAAGCATGCATACGGACATTATGTTATGCTACAACAATTGGAGCACA     564
cblA-1          TCGGAAACAGAAGACGGCATGCACTCCAGCTTCGAGGCTGTATACCGCAACTGGAGTACT     546
cepA-1          ATCCATACAGAAAACGATATGCATAAAAATCTGGAGTTCTGTTACCAAAACTGGACTACT     546
pbbA-1          GAAATGGATGAAAACGACATGCATGAGAATCTTGACAACTGTTACAGCAACTGGACTACA     543
cfxA-1          GCTTATACGGAAGAGGAAATGTCGGCTGACCATAACAAGGCTTACTCTAACTATACATCT     600
                         **       ***                     **    ** *  *   *
blaBAS-1        CCTTTGGCAGTGGCGGAACTATTGAAAAAGGTATACACAGAAGCCGTATTACCCGA---T     585
blaMUN-1        CCATTAGCTATTGCGCAATTGCTCAAGAAACTTCATACAGAAAATATTTTGACAAA---A     621
cblA-1          CCTTCCGCTATGGTCCGACTACTGAGAACGGCTGATGAAAAAGAGTTGTTCTCCAACAAG     606
cepA-1          CCATTAGCAGCCGCCAAATTACTGGAAATATTTCGCAATGAAAACCTTTTTGACAA---A     603
pbbA-1          CCTATGGCTGCTGTAAATCTAATGAATCTATTTCTTGACGGTGATGCAGTTTCCGG---T     600
cfxA-1          CCTCTTGGTGCTGCAATGTTGATGAATCGTTTGTTTACTGAAGGTCTTATCGATGA---T     657
                **    *     *      *  *                          *
blaBAS-1        GAGTATTTTAGATTCTTGGAAAAAGCAATGCTTGATTGTTCAACAGGAACCGACAAACTT     645
blaMUN-1        GAGCATTTTGCGTTTTTAGAGACAGCCATGCTTGATTGTGTGTCTGGCAAGAACAAACTA     681
cblA-1          GAGCTGAAAGACTTCTTGTGGCAGACCATGATAGATACTGAAACCGGTGCCAACAAACTG     666
cepA-1          GAATACAAGAATTTCATTTATCAAACAATGGTGGAATGTCAGACAGGACAAGACCGCCTG     663
pbbA-1          GAATATTATGATTTTATATATAGAACAATGACGGAATGTACTACGGGCACTGCCAGGCTG     660
cfxA-1          GAGAAACAAAGTTTCATTAAGAATACGTTAAAAGAATGCAAAACAGGTGTAGATAGGATA     717
                **          **  *        *  *    **        * **           *
blaBAS-1        AAAGCGGGACTGCCTAC------AGGTGTACGTTTTGGGCATAAAACGGGAAGTTCAGAC     699
blaMUN-1        ATAGCGGGGTTGCCTAC------CGATATAAAATTTGGGCATAAAACGGGACGTTCTAAC     735
cblA-1          AAAGGTATGTTGCCAGC------CAAAACCGTAGTAGGACACAAGACCGGCTCTTCCGAC     720
cepA-1          ATTGCTCCACTGCTCGATAA---AAAAGTAACAATGGGGCATAAAACCGGAACAGGCGAC     720
pbbA-1          CCGTATCCATTGAAAAACAC---TGGTGCCATAATAGGGCATAAGACAGGAACCGGTGAC     717
cfxA-1          GCAGCTCCACTTCTTGATAAAGAAGGGGTTGTTATAGCGCATAAGACAGGTTCAGGTTAT     777
                          *                       * *  ** ** ** **        *
blaBAS-1        CGTACAGAGATGGGGATTAAAATAGGTGATGGGGATGCCGGAGTGATTTATATGCCTGAT     759
blaMUN-1        CGAACTGCTGATGGCATACAGATAAGCGAGGGAGATGCAGGAGTGATATATCTTCCCAAC     795
cblA-1          CGCAATGCCGACGGTATGAAAACTGCAGATAATGATGCCGGCCTCGTTATCCTTCCCGAC     780
cepA-1          CGTAATGCGAAAGGACAACAGATCGGTTGCAATGACATCGGGTTTATTCTTCTTCCCGAC     780
pbbA-1          AAGAACTCCGATGGACGGTATATTGGCATAAATGATATCGGATTTATTGATTTGCCTAAT     777
cfxA-1          GTTAATGAAAATGGTGTTCTTGCAGCTCACAATGATGTTGCCTATATATGTCTGCCTAAT     837
                   *        **                   **    *      *     * **  *
blaBAS-1        GGCAGTAAATGTTATCTTGTAGTGATTATAAAGGATTCAAAAGAAACAGATGCCGAGAAT     819
blaMUN-1        GGTGAGAAGTGCTACATTGTCGTTTTGATAAAGGATTCTCGTGAGTCTGATGATGATAAC     855
cblA-1          GGCCGGAAATACTACATTGCCGCCTTCGTCATGGACTCATACGAGACGGATGAGGACAAT     840
cepA-1          GGACATGCCTATAGTATAGCCGTCTTCGTGAAAGATTCCGAAGCAGATAACAGAGAAAAC     840
pbbA-1          GGTGACCGTTATCTGATAGCTGTATTAGTGAAAGACTCTGCCGAGGATTATGAAACTACG     837
cfxA-1          AATATCAGTTATACCTTAGCGGTATTTGTTAAGGATTTCAAGGGAAATAAATCACAAGCG     897
                         *      * *  *   *  * *  ** *     *      *
blaBAS-1        GCAAAAATAATGGAGAATATCGCAAGTGCTGTCTATGATAGTTTAAAATAG---------     870
blaMUN-1        GCCAAAATAATGGCAGATATATCAAATATAGTTTATCGTCATTTGAATAAATAA------     909
cblA-1          GCGAACATCATCGCCCGCATATCACGCATGGTATATGATGCGATGAGATGA---------     891
cepA-1          AGTGAGATTATCGCAGAAATTTCGCGCATCGTTTACGAATACGTAACGCAACAGATAGAT     900
pbbA-1          GAAAGGATAATTGCTGAAATATCTTCTATGGTATATAAGAATATTAGTAATCAATAA---     894
cfxA-1          TCACAATATGTTGCGCATATATCAGCTGTAGTATATTCTTTATTAATGCAAACTTCAGTA     957
                          * *     **  *       ** **        * *
blaBAS-1        ---------       870
blaMUN-1        ---------       909
cblA-1          ---------       891
cepA-1          TAA------       903
pbbA-1          ---------       894
cfxA-1          AAATCTTAA       966
```

C)

BAS-1	100% (100%)					
MUN-1	64.01% (100%)	100% (100%)				
CblA-1	42.76% (99%)	42.20% (95%)	100% (100%)			
CepA-1	39.58% (97%)	41.03% (97%)	43.60% (97%)	100% (100%)		
PbbA-1	40.60% (97%)	39.11% (99%)	42.52% (98%)	51.82% (100%)	100% (100%)	
CfxA-1	31.11% (92%)	29.52% (88%)	29.39% (98)	38.02% (99%)	39.63% (97%)	100% (100%)
	BAS-1	MUN-1	CblA-1	CepA-1	PbbA-1	CfxA-1

Supplementary Figure 1. (A) Alignment of BAS-1, MUN-1, CblA-1, CepA-1, PbbA-1, and CfxA-1 amino acid sequences according to Ambler's numbering scheme. (B) Alignment of *bla*$_{\text{BAS-1}}$, *bla*$_{\text{MUN-1}}$, *cblA-1*, *cepA-1*, *pbbA-1*, and *cfxA-1* nucleotide sequences. Boldface amino acid residues are the highly conserved residues involved in the catalytic site of the class A β-lactamases. Asterisks indicate the identical residues in the β-lactamases. (C) Identity matrix of the alignment for the amino acid sequences. Reported values correspond to amino acid sequence identity, with alignment coverage provided in parentheses.

Supplementary Table 2. Methodology

<table>
<tr><th colspan="3">Antimicrobial susceptibility testing</th></tr>
<tr><td>Disk diffusion (OXOID)</td><td colspan="2">Cefazolin</td></tr>
<tr><td>Gradient strips (Liofilchem)</td><td colspan="2">Ampicillin, Amoxicillin, Amoxicillin-Clavulanic acid, Piperacillin, Piperacillin-Tazobactam, Cefazolin, Cefuroxime, Ceftazidime, Ceftazidime-Avibactam, Cefotaxime, Ceftolozane-Tazobactam, Cefepime, Ceftaroline, Aztreonam, Imipenem, Meropenem, Ertapenem</td></tr>
<tr><th colspan="3">Modified β-LIM assay</th></tr>
<tr><td colspan="3">1. Prepare Eppendorf tubes with 1 mL thioglycolate broth + vitamin K, a half 10 µL loop of bacterial biomass (BT162 and BT736), and cefazolin disks (60 µg).</td></tr>
<tr><td colspan="3">2. Set up one control (no inhibitor) and additional tubes, each with a different inhibitor. Fixed inhibitor concentrations:
- Clavulanic acid 2 mg/L,
- Tazobactam 4 mg/L,
- Avibactam 4 mg/L,
- Vaborbactam 8 mg/L,
- Relebactam 4 mg/L.</td></tr>
<tr><td colspan="3">3. Moreover, set up sterile negative control without bacteria encompassing 6 tubes:
1. thioglycolate broth (vitamin K) + cefazolin disks (60 µg)
2-6. thioglycolate broth (vitamin K) + cefazolin disks (60 µg) + inhibitors</td></tr>
<tr><td colspan="3">4. Add immersion oil to create anaerobic conditions and incubate for 24 h with shaking.</td></tr>
<tr><td colspan="3">5. Prepare a bacterial lawn of E. coli ATCC adjusted to 0.5 McFarland on Mueller-Hinton agar plate.</td></tr>
<tr><td colspan="3">6. Place cefazolin disks on the lawn, ensuring proper spacing, and incubate at 37 °C for 24 h.</td></tr>
<tr><th colspan="3">Software used for bioinformatic analysis</th></tr>
<tr><td rowspan="3">Assembly</td><td colspan="2">Guppy v5.0.0 (Super-Acurate basecalling)</td></tr>
<tr><td colspan="2">Flye 2.9.6</td></tr>
<tr><td colspan="2">Medaka 1.4.4</td></tr>
<tr><td rowspan="8">Genome analysis</td><td>Prokka 1.14.6</td><td>https://usegalaxy.org</td></tr>
<tr><td>ResFinder 4.7.2</td><td>https://genepi.food.dtu.dk/resfinder</td></tr>
<tr><td>PlasmidFinder 2.0.1</td><td>https://cge.food.dtu.dk/services/PlasmidFinder/</td></tr>
<tr><td>ANI Calculator</td><td>https://www.ezbiocloud.net/tools/ani</td></tr>
<tr><td>Clustal Omega</td><td>https://www.ebi.ac.uk/jdispatcher/msa/clustalo</td></tr>
<tr><td>iTOL v7.5.1</td><td>https://itol.embl.de</td></tr>
<tr><td>BLAST</td><td>https://blast.ncbi.nlm.nih.gov/Blast.cgi</td></tr>
<tr><td>SnapGene Viewer 8.2</td><td>https://www.snapgene.com</td></tr>
</table>

Supplementary Table 3. Quality metrics of the BT736 genome assembly

Contig	Lenght (bp)	Coverage	Circular	Repeat	Multip.	GC %
Contig 1	6072844	33	N	N	1	43
Contig 2	22415	111	Y	Y	4	40

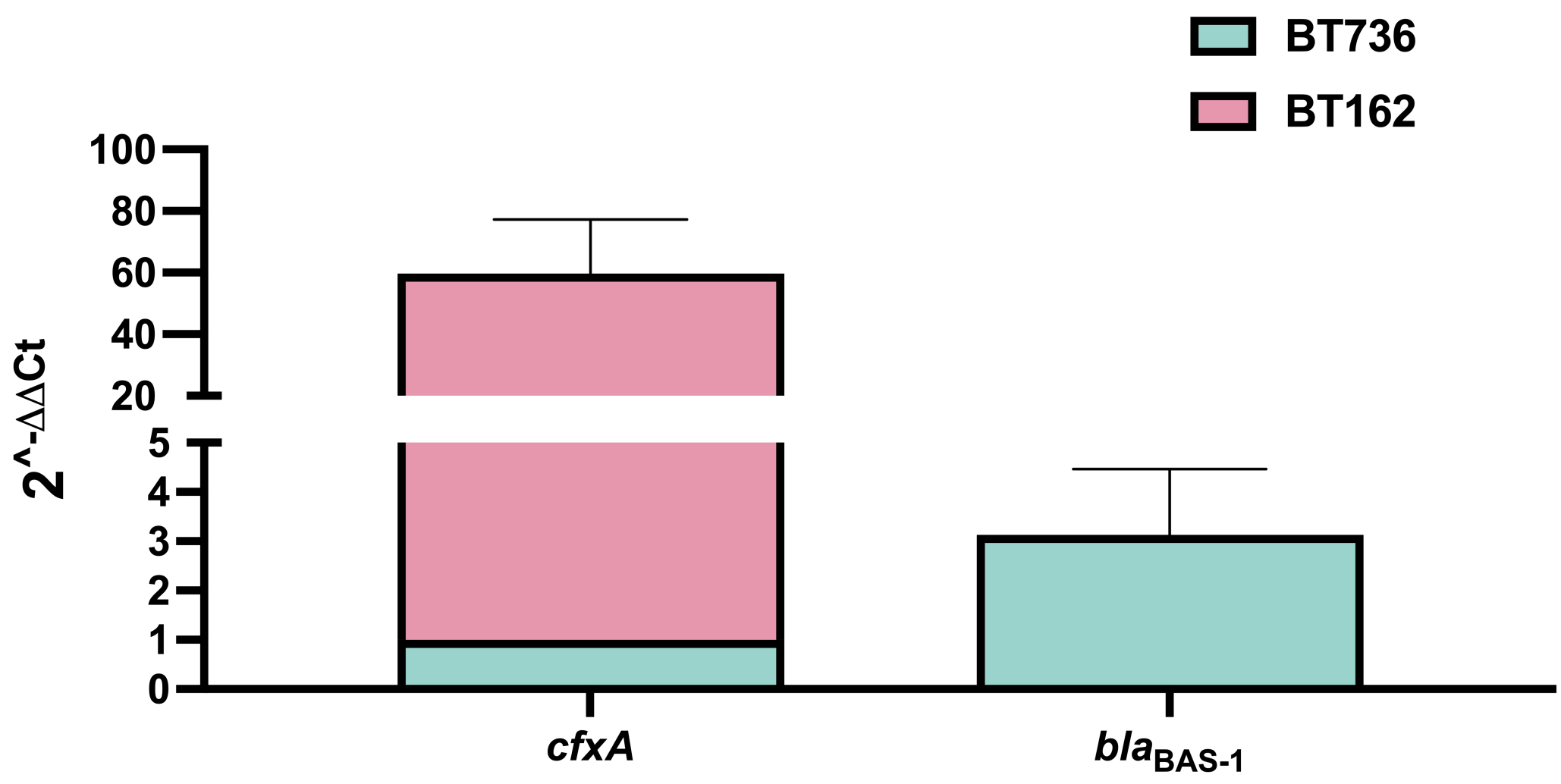

Supplementary Figure 2. *bla*$_{BAS-1}$ and *cfxA* gene transcription in the BT162 and BT736 isolates. Data were normalized to the mRNA levels of the 16S gene. Three independent replicates were performed.

Supplementary Table 4. IC_{50}s for β-lactamase inhibitors toward BAS-1

	**IC_{50} (μM)***		
**β-lactamase**	**Clavulanic acid**	**Tazobactam**	**Avibactam**
BAS-1	1.31	0.87	1.59

***IC_{50} : Represents the concentration of an inhibitor that is required for 50% inhibition of the enzymatic activity.**
